# Coverage Policies, Benefit Routes, and Public Financial Terms for Prescription Digital Therapeutics in the United States: A Cross-Sectional Health Policy and Market Access Study

**DOI:** 10.7759/cureus.117911

**Published:** 2026-10-14

**Authors:** Shaheen E Lakhan

**Affiliations:** 1 Research and Development, Boricua Bio, San Juan, USA; 2 Bioscience, Global Neuroscience Initiative Foundation, Boston, USA; 3 Neurology, Western University of Health Sciences, Pomona, USA; 4 Neurology, A.T. Still University School of Osteopathic Medicine in Arizona, Mesa, USA; 5 Medicine, Morehouse School of Medicine, Atlanta, USA; 6 Neurosciences, FIU Herbert Wertheim College of Medicine, Miami, USA

**Keywords:** digital medicine, formulary, insurance coverage, medical policy, pharmacy benefit, prescription digital therapeutics, price transparency

## Abstract

Background

Prescription digital therapeutics (PDTs) may be addressed through clinical policies, pharmacy benefits, purchaser-selected catalogs, or professional-care payment rules. A single covered/not-covered designation can obscure differences in scope, distribution, and financial responsibility. This study examined how these distinctions appear in a public-source corpus.

Methods

An exploratory cross-sectional health policy and market access study used an October 4, 2026 snapshot. Twenty-two regulatory-anchored therapeutic program families were classified as general-purpose-device programs or software-primary systems. Adaptive public-source searches assembled policy, benefit, program, and financial records. Operational product-scope statements were separated from general frameworks, historical documents, and manufacturer-reported access. Descriptive counts and sensitivity analyses were generated from source-linked data; national coverage prevalence and covered lives were not estimated.

Results

The dataset contained 35 operational source records. Twelve current-posted position documents from 10 publishing families supported 57 product-scope mappings across 21 of the 22 programs: 54 restrictive, two supporting conditional clinical eligibility, and one class-based mapping with unresolved delivery-setting applicability. Restrictive mappings comprised 45 medical/clinical-policy and nine pharmacy records; 48 were named and six class-derived. Anthem’s product-specific guideline contributed 15 restrictive mappings and addressed both listed applications and associated management services. Conditional clinical eligibility involved Luminopia (Luminopia, Inc., Cambridge, MA, USA) and CureSight (NovaSight Ltd., Airport City, Israel); the intravaginal biofeedback class mapped to leva (Axena Health, Inc., Newton, MA, USA) remained setting-uncertain. Deduplication across repeated family-product-route positions retained 53 mappings. The general-purpose-device-only analysis retained 30 restrictive mappings and excluded both conditional-eligibility mappings and the setting-uncertain mapping. Ten additional pathway records distinguished exception guidance, Medicare professional-care payment, optional digital catalogs, non-drug list architecture, and manufacturer reports. Fifteen financial observations were heterogeneous: a current-displayed $99 EndeavorRx (Akili Interactive Labs, Inc., Boston, MA, USA) prescription price, an expired $99 Rejoyn (Otsuka America Pharmaceutical, Inc., Rockville, MD, USA) offer, a $25 leva evaluation fee, and a $2,100 economic-model input were not interchangeable.

Conclusions

FDA authorization and billing codes did not establish favorable medical-necessity decisions in this corpus. Product-specific restrictions, exemplified by Anthem’s October 2026 guideline, coexisted with conditional pathways elsewhere. Clinical access assessment should verify the governing policy, plan adoption, benefit route, and patient cost before treatment. The broader product definition retained clinical-eligibility pathways and an uncertain class mapping excluded by the general-purpose-device-only analysis. These findings characterize selected documents, not national coverage prevalence or realized patient access.

## Introduction

Prescription digital therapeutics (PDTs) occupy an operational space between software, medical devices, professional care, and pharmacy administration. Regulatory authorization identifies a device and its intended use; it does not by itself establish the terms of an insurance contract. US Food and Drug Administration (FDA) databases are therefore necessary for product identification but insufficient to determine a patient’s financial access [1]. For stakeholders, the practical question is not only whether an intervention has been authorized, but also which policy applies, whether the relevant benefit includes it, how it is supplied, and what financial terms can be established before treatment begins.

Previous research has examined payer decision-making and Medicaid implementation. A qualitative study of 22 US payer decision-makers identified evidence requirements, benefit-policy adaptation, and billing as important considerations, with both medical and pharmacy routes contemplated [2]. Massachusetts Medicaid experience has informed value assessment and formulary management [3]. Separately, research has assessed limitations of the clinical evidence supporting PDTs [4]. These studies address related questions, but neither regulatory status nor an assessment of clinical evidence substitutes for extracting an operative coverage statement from a defined source.

Two measurement problems complicate public-policy research. First, “coverage” may refer to clinical eligibility, contractual benefit inclusion, a catalog that purchasers may select, an exception program, or payment for a clinician’s management service. Second, product definitions differ: a scope limited to general-purpose-device programs may omit therapeutic software delivered through a required headset, motion sensor, eye tracker, or wearable. Price comparisons are similarly vulnerable when product charges, promotional offers, professional fees, and modeled intervention costs are pooled. The objective of this cross-sectional health policy and market access study was to assemble a source-linked dataset that distinguishes these dimensions and tests how selected coding and product-scope choices change the descriptive findings. It was not designed to estimate national patient access or adjudicate whether a payer’s clinical conclusion was correct.

## Materials and methods

Study design and setting

This exploratory cross-sectional health policy and market access study analyzed publicly available US documents using an October 4, 2026 snapshot. Earlier document versions were examined where available for limited historical comparisons. Relevant Strengthening the Reporting of Observational Studies in Epidemiology (STROBE) domains guided reporting of selection, variables, missing information, and limitations; patient-level items were not applicable [5]. The study generated a coded observational dataset rather than pooling clinical treatment effects.

The study used purposive, adaptive selection to identify publicly available documents across delivery technologies, policy publishers, insurance settings, and financial source types. Source selection and coding were iterative, and coverage positions were available during development of the final codebook. The study was exploratory and was not designed to estimate national coverage prevalence.

Product definition and identification

The operational core comprised prescription products with a regulatory anchor in which software delivered a principal therapeutic intervention: structured cognitive or behavioral treatment, attention or motor training, visual retraining, biofeedback, or algorithm-guided therapeutic intervention. General-purpose-device programs delivered the intervention through a phone, tablet, or computer without requiring a therapeutic headset, dedicated sensor, eye tracker, or wearable. Programs requiring one of these components for the principal therapeutic function were classified as software-primary systems, including when the required hardware was commercially available rather than custom-built. The general-purpose-device-only analysis excluded those seven systems. Required hardware did not exclude a program from the broader study corpus. Systems whose principal intervention was mechanical assistance or electrical stimulation, diagnosis-only or monitoring-only tools, dose calculators, consumer wellness applications, and fertility-prediction software were outside this operational core.

The study corpus comprised 15 general-purpose-device programs and seven software-primary systems identified through regulatory database and product-source checks [1]. FDA authorization identifiers, source URLs, regulatory names, clinical domains, aliases, and scope notes were retained for all 22 program families (Table 1). Discovery used manufacturer and regulatory sources and condition-specific policy leads; it was not an independent FDA census. A program-family identifier prevented duplicate counting of aliases but did not establish equivalence of all software versions. Consumer programs were not substituted for similarly named prescription products. Policy claims were restricted to the indication under discussion; for example, a urinary-incontinence policy was not applied to fecal incontinence.

**Table 1:** Regulatory-anchored program corpus The 22 programs form a selected study corpus, not an exhaustive FDA or active-market census. Manufacturer/applicant names and locations reflect the cited regulatory anchors; Freespira and leva use the current manufacturer information in the cited product sources. These details do not establish current ownership, commercial supply, or equivalence of every marketed version. FDA authorization identifiers identify the source records available through the FDA device databases [1]. FDA anchors are not necessarily the latest label; full labeling governs. leva’s conditional mapping concerns urinary incontinence only. reSET-O is a treatment-retention adjunct; no abstinence claim is inferred. US postal abbreviations denote states. FDA, US Food and Drug Administration; ADHD, attention-deficit/hyperactivity disorder; IBS, irritable bowel syndrome; PTSD, posttraumatic stress disorder; USA, United States of America

Program family	FDA anchor	Clinical domain (summary)	Delivery stratum
AspyreRx (Better Therapeutics, San Francisco, CA, USA)	DEN220058	Type 2 diabetes	General-purpose
CT-132 (Click Therapeutics, Inc., New York, NY, USA)	DEN240064	Episodic migraine prevention	General-purpose
Motivista/CT-155 (Click Therapeutics, Inc., New York, NY, USA)	K254038	Negative symptoms of schizophrenia	General-purpose
DaylightRx (Big Health, Inc., San Francisco, CA, USA)	K233872	Generalized anxiety disorder	General-purpose
EndeavorRx (Akili Interactive Labs, Inc., Boston, MA, USA)	K231337	ADHD-related attention function	General-purpose
LumosityRx (Lumos Labs Medical, a division of Lumos Labs, Inc., San Francisco, CA, USA)	K243729	ADHD-related attention function	General-purpose
MamaLift Plus (Curio Digital Therapeutics, Inc., Princeton, NJ, USA)	K223515	Mild-to-moderate postpartum depression	General-purpose
Parallel/Mahana IBS (Mahana Therapeutics, Inc., San Francisco, CA, USA)	K211372	Irritable bowel syndrome	General-purpose
Regulora (MetaMe Health, Inc., Chicago, IL, USA)	K211463	IBS-associated abdominal pain	General-purpose
Rejoyn (Otsuka America Pharmaceutical, Inc., Rockville, MD, USA)	K231209	Major depressive disorder	General-purpose
reSET (Pear Therapeutics, Inc., Boston, MA, USA)	DEN160018	Specified substance-use-disorder population	General-purpose
reSET-O (Pear Therapeutics, Inc., Boston, MA, USA)	K173681	Opioid-use-disorder outpatient treatment retention	General-purpose
SleepioRx (Big Health, Inc., San Francisco, CA, USA)	K233577	Chronic insomnia/insomnia disorder	General-purpose
Somryst (Pear Therapeutics, Inc., San Francisco, CA, USA)	K191716	Chronic insomnia	General-purpose
Stanza (Swing Therapeutics, Inc., San Francisco, CA, USA)	DEN220083	Fibromyalgia symptoms	General-purpose
Luminopia (Luminopia, Inc., Cambridge, MA, USA)	DEN210005	Amblyopia	System/accessory
CureSight (NovaSight Ltd., Airport City, Israel)	K221375	Amblyopia	System/accessory
Freespira (Freespira, Inc., Kirkland, WA, USA)	K233337	Panic symptoms/PTSD	System/accessory
RelieVRx (AppliedVR, Inc., Van Nuys, CA, USA)	DEN210014	Chronic lower back pain	System/accessory
leva (Axena Health, Inc., Newton, MA, USA)	K213913	Urinary/fecal incontinence	System/accessory
NightWare (NightWare, Inc., Hopkins, MN, USA)	DEN200033	Nightmare-related sleep disturbance	System/accessory
MindMotion GO (MindMaze SA, Lausanne, Switzerland)	K173931	Motor rehabilitation	System/accessory

Commercial supply was not uniformly verified. The primary denominator is therefore a regulatory-anchored program corpus, not all actively obtainable PDTs. Availability signals, including a waitlist, were recorded when directly observed.

Sources, retrieval, and source hierarchy

Source discovery combined public web searches with direct review of official policy portals, regulatory databases, and linked documents. Where original financial-source URLs redirected to general pages, direct supporting sources were identified during revision to corroborate the previously extracted observations; their revision access dates are reported in the references.

Searches were organized around class, product, indication, and publisher. Key concepts included PDTs, digital health technologies, therapeutic applications, mobile device-based health management, dichoptic therapy, amblyopia, and biofeedback. Brand names and aliases in Table 1 were paired with coverage, medical policy, pharmacy, formulary, prior authorization, price or cost, and searches were restricted to publisher domains when appropriate. Representative query templates were: site:[publisher domain] "prescription digital therapeutics", site:[publisher domain] "[product or alias]" "medical policy", and site:[manufacturer domain] "[product]" cost. These templates describe how to repeat the targeted approach; they are not a verbatim log of every original query. The complete query sequence and search-result screening counts were not retained.

The primary position corpus included Aetna [6], Cigna [7], Anthem/Elevance [8-10], Blue Cross and Blue Shield of North Carolina (Blue Cross NC) [11], Premera Blue Cross [12], Blue Cross Blue Shield of North Dakota (BCBSND) [13], Blue Cross & Blue Shield of Mississippi (BCBSMS) [14], Blue Cross & Blue Shield of Rhode Island (BCBSRI) [15], a MyHealthToolkit publishing-family policy [16], and UnitedHealthcare sources [17,18]. A publishing family was an organizational grouping of the observed sources, not proof that all affiliated plans used identical criteria. New York- and Virginia-hosted Anthem guidelines remained separately scoped within one publishing family. The MyHealthToolkit page was not assigned to a particular insurer without adoption evidence.

Anthem sources were distinguished by role: CG-ANC-08 supplied a general framework [8], CG-ANC-11 supplied named-product determinations [9], and CG-MED-102 supplied amblyopia criteria [10]. Only products meeting the study definition contributed mappings. Plan-adoption caveats and document history informed interpretation; publication of a new guideline was not assumed to represent a new coverage decision.

Additional layers included a Highmark Wholecare Medicaid exception-guidance notice [19], Centers for Medicare & Medicaid Services (CMS) professional-care payment rules for Medicare [20], Evernorth’s digital catalog [21], and the MassHealth non-drug list portal [22]. A historical Highmark commercial policy [23] and an embedded earlier BCBSND version [13] were analyzed separately from current positions. Manufacturer access statements were never upgraded to payer-verified routine benefits.

For inclusion in the current position corpus, an official issuing organization had to provide an accessible operative statement applicable to a program meeting the study definition, with an identifiable population or policy scope and a document version applicable to the snapshot. Either a named-product statement or a class statement with a defensible product-and-indication match could qualify. Background discussion, a search-result snippet, a billing-code listing alone, and a manufacturer claim were insufficient to establish a payer position. Historical versions, general frameworks, exception announcements, purchaser catalogs, and financial or identity materials were retained in their separate evidence layers.

Selection proceeded by opening potentially relevant policy and product leads, following links to related or more specific official documents, checking product identity and indication, and extracting the controlling statement together with its scope and date. Repeated access to the same document was not counted as a new document; distinct populations within one document could generate separate scope records. Documents with unavailable operative text or unresolved currency could not establish a current position. The final set was purposively assembled for the stated descriptive analysis, without a fixed national sampling frame or a claim of search saturation.

The included English-language sources are identified by publisher, document title or identifier, URL, and dates in the references. The source ledger additionally records the supporting page or section, retrieval mode, and unresolved qualifications. This permits re-extraction of included records, although the missing original query log prevents exact reproduction of the discovery and exclusion sequence.

Coverage, benefit, and pathway variables

After source selection, each operative position record linked a program to a source-specific population, route, and date. “Restrictive” required an explicit experimental, investigational, unproven, not-medically-necessary, or exclusion statement. “Conditional clinical” identified an affirmative clinical-eligibility pathway under stated criteria; a setting-related ambiguity was retained as a separate conditional-uncertain category. Evidence specificity distinguished named negative determinations, class-derived mappings, and an affirmative class that named product examples. A restrictive clinical policy was not automatically coded as exclusion through every benefit.

Benefit inclusion was recorded separately. Explicit pharmacy withdrawal or commercial contractual exclusion was retained as such; clinical criteria with a member-contract caveat did not establish routine benefit inclusion. General class frameworks, optional purchaser catalogs, program notices, and manufacturer-reported access were stored in a separate pathway table rather than duplicated across all 22 programs. An absent product-policy mapping means no operative mapping was extracted in this corpus, not non-coverage or a completed payer-wide negative search. Distribution and payment route were kept separate. Where a specific applicable policy controlled a general guideline, the specific statement determined the operative record.

Restrictions were captured only when stated, including prior treatment, age, testing, concurrent care, course duration, renewal, and setting. Silence was not coded as absence of a requirement. A source’s definition of an appropriate treatment population was recorded without assuming that narrower eligibility was clinically unjustified. When policies described treatment as unproven, we also recorded any stated coverage exceptions or provisions for individualized medical-necessity review.

Financial observations

To assess the financial terms accompanying these pathways, financial extraction distinguished advertised product cash prices, promotions, displayed reference amounts, clinical-visit fees, model inputs, manufacturer-reported patient costs, and payment-code rules. Sources included EndeavorRx (Akili Interactive Labs, Inc., Boston, MA, USA) [24], Rejoyn (Otsuka America Pharmaceutical, Inc., Rockville, MD, USA) [25], leva (Axena Health, Inc., Newton, MA, USA) [26,27], Freespira (Freespira, Inc., Kirkland, WA, USA) [28], and RelieVRx (AppliedVR, Inc., Van Nuys, CA, USA) [29]. An undated amount displayed on a current page was not equivalent to a completed transaction or payer-allowed amount. An explicitly expired offer could not be classified as a current promotion. Missing numerical amounts remained missing, not zero.

Units and package conditions were retained rather than converted to an assumed annual cost. Professional fees were not added to or substituted for product prices without evidence that they formed the same package. A modeled intervention cost was not relabeled as wholesale acquisition cost. Manufacturer-reported zero additional cost for an eligible program was retained as a conditional claim, not a payer-verified universal benefit. Because a uniform price search for every program was not completed, the study did not estimate the marketwide proportion with public prices or calculate an average PDT price.

Analysis and quality controls

Descriptive counts and tables were generated in Microsoft Excel (Microsoft Corporation, Redmond, WA, USA). Current and historical positions were separated. Descriptive outputs counted documents, publishing families, program-scope mappings, routes, and source specificity. The same BCBSRI statement applied to two separately stated insurance scopes and was counted at that scope level, while a sensitivity analysis collapsed repeated family-product-route-position records. The general-purpose-device-only analysis restricted the corpus by the delivery rule defined above; a named-only sensitivity excluded all class-based mappings. These were exploratory sensitivity analyses, not pre-specified confirmatory tests.

The study did not perform significance testing, regression, population weighting, or a power calculation. Program-policy cells are not independent subjects, and the purposive corpus cannot support an unbiased national prevalence estimate. Public organizational documents were used without patient-level or identifiable private records; no institutional ethics determination is asserted. Figure 1 summarizes the methodological framework, including the separation of current policy positions from other evidence layers.

**Figure 1:**
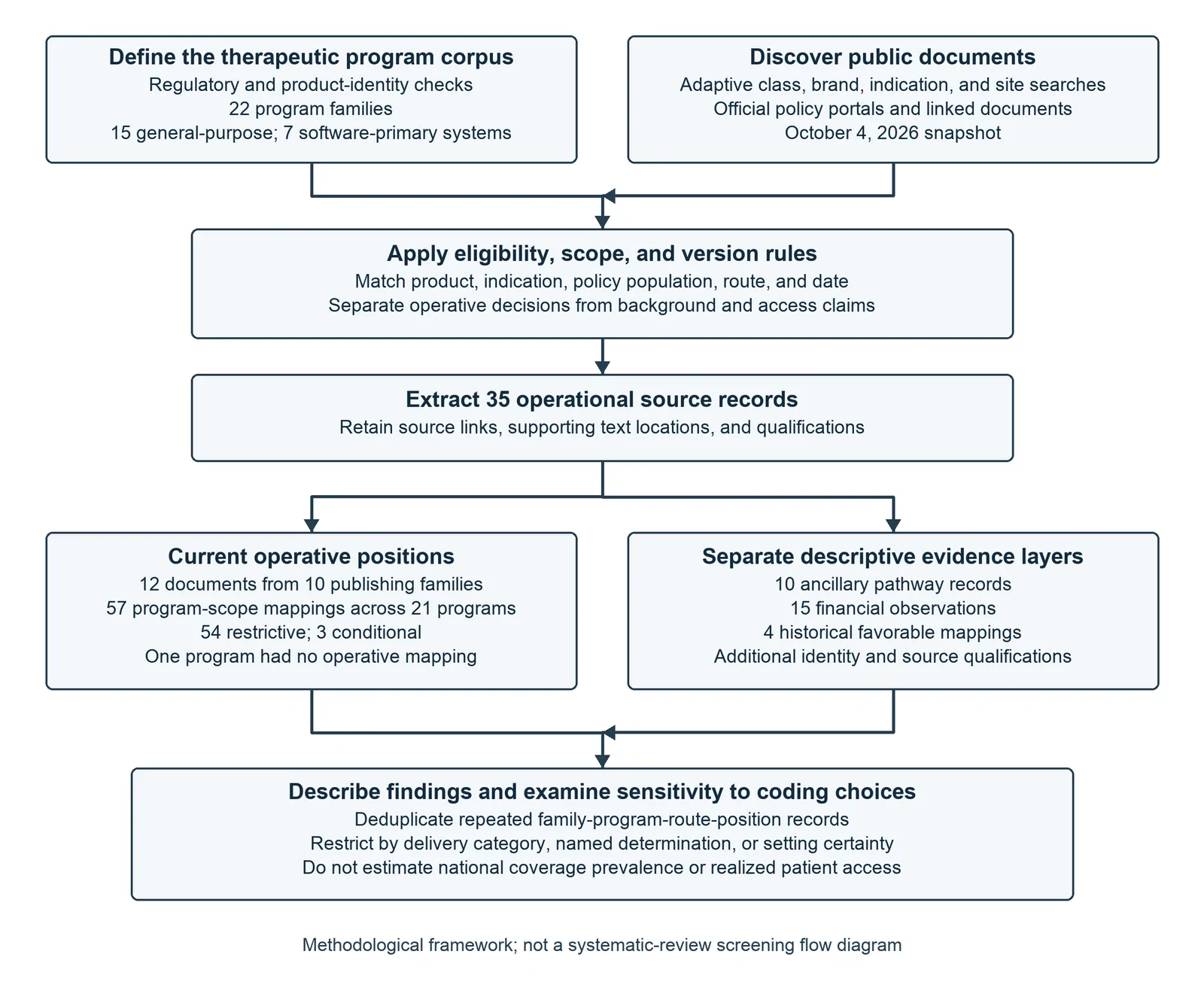
Methodological framework for the cross-sectional health policy and market access study The diagram summarizes the executed purposive, adaptive workflow. Arrows describe analytic relationships rather than a chronological screening census; product identification, source selection, and coding were iterative. Source discovery was anchored to October 4, 2026. The 35 operational source records are not 35 independent current policy documents. The three records in the broader conditional category comprise two supporting conditional clinical eligibility and one class mapping with unresolved setting applicability. Evidence layers may draw on the same source, so the 10 pathway records, 15 financial observations, and four historical mappings should not be summed as mutually exclusive document counts. The complete original search-result screening counts were not retained. FDA: US Food and Drug Administration

## Results

Corpus and program breadth

The dataset contained 22 program families: 15 general-purpose-device programs and seven software-primary systems (Table 1). It comprised 35 operational source records covering current policies, historical versions, benefit and payment frameworks, manufacturer access reports, and financial or identity materials. Twelve current-posted position documents from 10 publishing families generated 57 operative program-scope records covering 21 of the 22 programs. No operative product-specific mapping was extracted for Motivista (CT-155), and its inclusion did not add a restrictive or conditional record. This absence does not establish noncoverage. A separate general guideline supplied framework information but did not constitute an additional named position document [8].

Commercial medical-policy sources [6,7], a commercial pharmacy notice [11], an Ohio Medicaid managed-care policy [18], a mixed commercial/Medicaid-expansion policy [13], and separate Medicare Advantage and commercial clauses [15] were represented. This breadth was purposive; neither all policies from those organizations nor all participating insurance plans were assessed. Table 2 reports the exact count contribution of each publishing family, and the sensitivity analyses.

**Table 2:** Current-posted operative position corpus The 22-program corpus yielded operative mappings for 21 programs. No operative mapping was extracted for Motivista (CT-155); this is not evidence of noncoverage. Unit: program × source-specific scope × route × position. Forty-five of 54 restrictive mappings are medical/clinical-policy records. BCBSRI has two scopes for the same two products; UnitedHealthcare has two amblyopia-class scopes. The broader conditional category includes two class-with-named-example mappings supporting clinical eligibility and one setting-uncertain class mapping; it does not mean three affirmative coverage decisions. BCBSMS counts reflect its general policy statements, subject to the Federal Employee Program (FEP) exception; no FEP coverage determination was inferred [14]. Counts are not insurer coverage percentages. General frameworks and manufacturer reports are not counted here. Premera denotes Premera Blue Cross. BCBSMS, Blue Cross & Blue Shield of Mississippi; BCBSND, Blue Cross Blue Shield of North Dakota; BCBSRI, Blue Cross & Blue Shield of Rhode Island; Blue Cross NC, Blue Cross and Blue Shield of North Carolina

Publishing family	Documents	Restrictive mappings	Conditional mappings	Pharmacy mappings	Unique programs
Aetna [6]	1	14	0	0	14
BCBSMS [14]	1	2	0	0	2
BCBSND [13]	1	0	1	0	1
BCBSRI [15]	1	4	0	0	2
Blue Cross NC [11]	1	9	0	9	9
Cigna [7]	1	2	0	0	2
Elevance guideline family [9,10]	2	15	2	0	17
MyHealthToolkit publishing family [16]	1	2	0	0	2
Premera [12]	1	2	0	0	2
UnitedHealthcare [17,18]	2	4	0	0	2
Total	12	54	3	9	21 unique
Sensitivity analysis					
Analytic view		Restrictive	Conditional	Interpretation	
Primary scope-level records		54	3	57 source-linked records	
Family-product-route-position deduplicated		50	3	53; four duplicated scopes collapsed	
General-purpose-device-only analysis		30	0	Two conditional-eligibility mappings and one setting-uncertain mapping excluded	
Explicitly named determinations only		48	0	Excludes class-based and class-with-named-example pathways	
Exclude setting-uncertain leva mapping		54	2	One ambiguous delivery-setting mapping removed	

Restrictive positions and route-specific scope

Of the 57 current operative mappings, 54 were restrictive: 48 named determinations and six class-derived mappings. Forty-five arose from medical or clinical-policy sources and nine from the pharmacy notice. Aetna contributed 14 named restrictive mappings [6]; Cigna contributed two [7]; the specific Anthem guideline contributed 15 [9]; Blue Cross NC contributed nine [11]; Premera contributed two class mappings [12]; BCBSMS contributed two under its general policy statements [14]; BCBSRI contributed four scope-specific mappings concerning two products [15]; MyHealthToolkit contributed two [16]; and UnitedHealthcare contributed four class mappings across commercial/exchange and Ohio Medicaid scopes [17,18]. These figures count documentary mappings, not independent insurer votes or coverage rates. The BCBSMS Freespira and NightWare mappings reflect its general investigational statements. Its Federal Employee Program (FEP) exception may require assessment of FDA-approved devices on medical necessity rather than investigational status. The two mappings therefore should not be generalized to FEP members; the exception itself does not establish coverage [14].

The Blue Cross NC notice concerned withdrawal of nine in-scope products from its specified pharmacy population, including two systems excluded from the general-purpose-device-only analysis. Its population exceptions and unresolved medical route were preserved [11]. BCBSRI’s same document provided distinct explanations for Freespira and NightWare (NightWare, Inc., Hopkins, MN, USA): insufficient evidence under Medicare Advantage and contractual exclusion under commercial products. Its four scope mappings therefore reflect two products, two scopes, and one document [15]. UnitedHealthcare’s Ohio source retained the state-specific medical-necessity review proviso rather than supporting an exception-free noncoverage conclusion [18].

Anthem: named-product restrictions and associated services

Anthem’s Selected Mobile Device-Based Health Management Applications guideline (CG-ANC-11) was the largest single-document contributor, accounting for 15 of the 54 restrictive mappings. It classified its listed applications as “not medically necessary for any indication” [9]. The study count includes only program families meeting the operational definition, rather than every application named in the guideline.

Its coding section addressed device supply and onboarding (G0552) and associated treatment-management services (G0553/G0554) when linked to the listed applications [9]. These service-code statements did not create additional program mappings. They showed why the availability of a professional-care billing code could not be treated as an affirmative product-level payment determination.

The October 1, 2026 publication followed transfer of product-specific content from CG-ANC-08; the new document date did not establish a new restriction for every listed product. Adoption could vary by plan or line of business, and applicable law, contract language, and medical policy took precedence over the clinical guideline [8,9]. The extracted determinations therefore describe the reviewed guideline, not universal exclusion across all Anthem-affiliated plans.

Conditional eligibility and policy hierarchy

Three mappings formed the broader conditional category: two supported conditional clinical eligibility, and one had unresolved delivery-setting applicability. Anthem’s amblyopia guideline named Luminopia (Luminopia, Inc., Cambridge, MA, USA) and CureSight (NovaSight Ltd., Airport City, Israel) as examples within an affirmative clinical framework [10]. BCBSND’s intravaginal motion-sensor biofeedback provision supported a class-to-leva mapping for urinary incontinence, but the separate exclusion of unsupervised home biofeedback left setting applicability unresolved [13]. None of these mappings established routine benefits for every plan member.

For amblyopia, the guideline required an age range, relevant diagnosis, prior conventional treatment, and specified functional/safety conditions. It permitted an initial 12-month interval and conditioned renewal on adherence and continued visual improvement. The eye-tracking performance requirement applied to CureSight; exact product-label overlap still requires attention [10]. BCBSND’s general urinary-incontinence biofeedback criteria described prior pelvic-floor exercise failure, but their relationship to the separate intravaginal provision should not be silently resolved [13]. Table 3 summarizes these conditional criteria alongside the ancillary access pathways.

**Table 3:** Conditional and ancillary pathways CG-MED-102, Y-20-014, and MM14315 are source document identifiers; G0552 is a payment code. Conditional clinical eligibility and manufacturer-reported access do not establish routine coverage for every member. BCBSND, Blue Cross Blue Shield of North Dakota; CMS, Centers for Medicare & Medicaid Services; DMHT, digital mental health treatment; FDA, US Food and Drug Administration; PDT, prescription digital therapeutic

Source/layer	Observed pathway	Conditions and unresolved elements
Anthem CG-MED-102 [10]	Luminopia and CureSight: conditional clinical eligibility	Specified amblyopia/age, prior treatment, functional/safety criteria; initial 12 months; adherence and improvement for renewal. Member benefit and product-label overlap still govern.
BCBSND Y-20-014 [13]	Intravaginal motion biofeedback: class-to-leva mapping	Urinary incontinence only; home supervision and relationship of criteria remain uncertain. No named product benefit established.
Highmark Wholecare [19]	Somryst exception guidance announced	Pennsylvania Medicaid notice; underlying full criteria not obtained. Not routine formulary inclusion.
CMS MM14315 [20]	Qualifying DMHT professional-care pathway	Appropriate FDA class and prescription use, practitioner-incurred device cost, ongoing behavioral care. G0552 contractor pricing is not a national product price.
Evernorth [21]	Optional purchaser-selected digital catalog	Catalog listing does not establish any particular client’s election or prescription-product inclusion.
MassHealth portal [22]	Preferred and other non-drug list architecture	Current named PDT placement and tier not retrieved; do not infer inclusion from portal existence.
Luminopia/CareFirst [30]	Manufacturer-described medical workflow with pharmacy fulfillment	Payer corroboration absent; prescription/authorization support does not prove all-member coverage.

Cigna’s explicit Freespira and RelieVRx exclusions remained operative despite its general conditional pathway. Its broader background language introduced uncertainty for interpreting that general pathway but did not erase the named determinations [7]. Anthem’s specific application guideline displaced a framework-only interpretation for 15 in-scope programs [8,9]. Thus, the presence of a general policy was not evidence of affirmative named-product coverage.

Formulary, program, and professional-care pathways

Ten ancillary pathway records supplemented the operative policy determinations: two general class frameworks, one exception announcement, one federal payment framework, one purchaser catalog, one non-drug formulary architecture, and four manufacturer-reported access records. Highmark Wholecare’s notice announced Program Exception guidance for Somryst (original manufacturer: Pear Therapeutics, Inc., San Francisco, CA, USA), but full operative criteria were not retrieved [19]. Evernorth described purchaser selection of individual digital solutions, not default coverage for every client [21]. MassHealth’s portal distinguished non-drug and preferred non-drug list routes; current named-product placements were not established from the downloaded material available in this audit [22].

CMS’s 2026 summary described a conditional professional-care route for qualifying FDA device classes, including an expansion to attention-deficit/hyperactivity disorder (ADHD). The practitioner must incur the device cost; G0552 is contractor-priced, while related management services are nationally priced. No brand-level reimbursement conclusion or numerical national supply rate was derived [20]. A manufacturer description of Luminopia’s CareFirst pathway involved pharmacy fulfillment with medical-benefit authorization, illustrating why those variables must remain distinct. The payer-specific coverage assertion was not independently corroborated and was not included in the primary position counts [30].

Financial terms and historical checks

Fifteen financial observations comprised 13 program-specific records across eight programs and two class-level Medicare records; seven contained a numerical amount (Table 4). Only one was a current-displayed, nonexpired product cash quote in the selected financial corpus: EndeavorRx at $99 per 30-day prescription [24]. The Rejoyn page still displayed a $99 promotion and $220 reference amount, but its offer terms ended June 30, 2026, and restricted reimbursement. The promotion was therefore expired at the snapshot, and the reference amount was not treated as a verified current regular price [25].

**Table 4:** Public financial observations The 14 data rows represent all 15 financial observations: 13 program-specific records across eight programs and two class-level Medicare records. The Freespira eligible-member cost row groups two observations; every other data row represents one. The n column counts dataset observations, not independent products or comparable prices. Non-numeric rows are missing or conditional information, not zero prices. Freespira’s eligible-member cost statements are manufacturer reports [31,32]; the LumosityRx waitlist [33] and unresolved SleepioRx comparison [34] do not establish current product prices. No pooled average, annualization, or price-availability prevalence is reported. CMS, Centers for Medicare & Medicaid Services; USFHP, US Family Health Plan; WAC, wholesale acquisition cost. G0552, G0553, and G0554 are payment codes

Program/source	Observations, n	Amount and unit	Classification/limitation
EndeavorRx [24]	1	$99 per 30-day prescription	Current-displayed product cash price; no expiry found. Not a payer rate or transaction.
Rejoyn [25]	1	$99 promotional course	Explicit expiration June 30, 2026: not a current verified offer. Cash reimbursement restrictions apply.
Rejoyn [25]	1	$220 displayed reference	Not established as current regular price or WAC.
leva [26]	1	$25 virtual evaluation	Professional visit fee; not therapeutic product price.
leva [27]	1	$2,100 modeled intervention	Economic-model input; not a current market quote.
Freespira [31,32]	2	$0 additional cost claimed for eligible USFHP/Veteran programs	Two conditional manufacturer representations; payer benefit not independently corroborated.
Freespira [28]	1	Individualized terms; no uniform amount extracted	Plan-dependent access/quotation.
RelieVRx [29]	1	Three-month rental; numeric charge not extracted	Rental/access term differs from treatment-session schedule.
LumosityRx [33]	1	No numeric quote; waitlist displayed	Consumer Lumosity subscriptions not substituted.
SleepioRx [34]	1	No qualified numerical amount coded	Zero-cost comparison context insufficient for a universal $0 interpretation.
Luminopia [35]	1	No qualified numerical product price extracted	Reviewed product-access page; not a comprehensive search of all contracts or suppliers.
Freespira, employer quotation [36]	1	Custom purchaser terms; no numerical amount extracted	Quote request does not establish employer adoption or a unit price.
CMS G0552 [20]	1	Contractor-priced supply/onboarding	No uniform national product charge extracted. Management payments distinct.
CMS G0553/G0554 [20]	1	Nationally priced management services; no numerical amount extracted	Professional management payment is distinct from device supply and acquisition cost.
Total	15	13 program-specific + 2 class-level Medicare observations	Eight named programs; seven observations include a numerical amount.

The $25 amount in leva’s prescription pathway was a virtual-evaluation fee, not its product price [26]. The $2,100 intervention amount described in an economic-model announcement was a model input, not a current payer payment or retail quotation [27]. Freespira’s selected pages described plan-dependent terms [28], while RelieVRx described a three-month rental arrangement without a numerical charge in the inspected page [29]. Freespira advertised zero additional cost for eligible US Family Health Plan members at Brighton Marine [31] and eligible veterans using its described Community Care Network pathway [32]; these were manufacturer statements, not independently verified universal benefits. These observations could not support a pooled price range or mean.

LumosityRx (Lumos Labs Medical, a division of Lumos Labs, Inc., San Francisco, CA, USA), FDA-cleared under the device name Prismira, is a prescription digital treatment for ADHD-related attention impairment. Its page displayed a waitlist without a current product price [33]. SleepioRx (Big Health, Inc., San Francisco, CA, USA) is a prescription digital treatment for insomnia. Incomplete eligibility context for its zero-cost comparison was retained as an unresolved financial observation, not a universal $0 price [34]. Consumer subscriptions were not substituted for either prescription product.

Luminopia’s reviewed product-access page yielded no qualified numerical product price [35]. A separate Freespira employer-quotation record likewise contained no numerical amount [36]. The two class-level Medicare observations concerned G0552 supply/onboarding and G0553/G0554 management services, respectively [20]. Table 4 reports the observation count for each row.

Four historical favorable mappings were kept outside the current totals: three from the unreconciled 2024 Highmark commercial policy [23] and one from an earlier BCBSND Freespira provision [13]. The latter was omitted from the April 2026 criteria [13]. That documented wording change was not interpreted as proof of universal benefit withdrawal. Likewise, the Highmark source’s conditional language and administrative services only (ASO) election caveat were not carried forward to a 2026 current-benefit claim without version reconciliation [23].

Sensitivity analyses

Collapsing duplicate publishing-family, program, route, and position combinations reduced 57 current scope records to 53, including 50 restrictive mappings, two supporting conditional clinical eligibility, and one with unresolved setting applicability. The four-record reduction reflected repeated BCBSRI scopes and UnitedHealthcare scopes. The general-purpose-device-only analysis retained 30 restrictive mappings and excluded the two conditional-eligibility mappings and the setting-uncertain class mapping. Restriction to explicitly named determinations retained 48 restrictive mappings and excluded the conditional class/example observations. Excluding the setting-uncertain leva mapping left two conditional mappings. These sensitivities demonstrate dependence on measurement choices; they do not establish that a delivery technology causes coverage or that class-based pathways are invalid.

## Discussion

Principal findings

This study shows why PDT coverage cannot be adequately described by a single covered-or-not-covered label. Within the selected documents, restrictive clinical policies and pharmacy exclusions coexisted with conditional clinical eligibility, exception processes, and other potential access arrangements. These statements answer different questions. Meeting a policy’s clinical criteria does not establish that a patient’s benefit includes the product, and a potential payment pathway does not establish that treatment was authorized or paid. The study therefore describes what the documents said, rather than how many patients could obtain treatment. Interpreting these findings requires attention to both the product definition and the applicable policy.

Product selection also changed the findings. The general-purpose-device-only analysis excluded the two conditional-eligibility mappings and the setting-uncertain leva class mapping found in the broader study. This does not show that software delivered through dedicated devices or accessories is more likely to be reimbursed; the products, indications, and policies were not independently sampled. It shows that a definition limited to general-purpose-device programs can miss relevant policy statements, including class criteria whose product-level applicability remains uncertain. Studies should define products by their therapeutic function and report delivery categories separately. Counting only explicitly named products can also miss potentially relevant class-based criteria, although those criteria require careful product-level verification. The Anthem findings illustrate how these measurement choices affect interpretation within a single publishing family.

Why the Anthem findings matter

The Anthem findings make the consequences of policy hierarchy concrete. One October 2026 guideline generated 15 restrictive mappings [9], while a separate amblyopia guideline supported two conditional mappings within the same publishing family [10]. A single insurer-level label would hide that difference. The appropriate unit of interpretation is the product, indication, applicable policy, and plan, rather than the organization’s overall approach to digital health.

For clinical access, regulatory authorization, medical necessity, and payment are separate decisions. A billing code identifies a service that can be described on a claim; it does not establish that the payer will accept the underlying treatment. The Anthem findings therefore caution against treating a professional-service route as a solution to an applicable product-specific restriction. Medicare payment provisions concern a federal professional-care payment framework [20], whereas the Anthem guideline provides clinical determinations subject to plan adoption [9]. Their different coverage settings prevent one from establishing the other’s payment decision.

For manufacturers, the practical implication is to identify the intended payer’s evidence requirements and benefit route during development, then determine which questions remain after regulatory authorization. For clinicians, it is to establish whether the patient’s plan adopts the product-specific guideline before arranging treatment or estimating patient costs. These recommendations follow from the documented separation of regulatory, clinical-policy, and payment decisions; they do not establish that Anthem’s assessment of any individual trial or product was correct.

Implications for clinical practice

Before prescribing, clinicians and access teams should verify the exact product, version, and intended use. They should then identify the current policy for the patient’s plan and determine whether the request falls under the medical benefit, pharmacy benefit, or a separate program. The next step is to check clinical eligibility, prior authorization, required prior treatment, supervision, and treatment or renewal limits. Finally, they should confirm the supplier, current product availability, and the patient’s expected charges. Unresolved benefit or cost questions should be documented rather than presented to the patient as confirmed coverage.

The distinction between benefit route and distribution is particularly important. The pharmacy withdrawal observed in this study did not establish exclusion through every other benefit [11], while a manufacturer-described pathway combined pharmacy fulfillment with medical-benefit authorization [30]. Neither observation establishes coverage for an individual patient. When a restrictive policy also specifies an exception process or individualized medical-necessity review, both should be recorded and their applicability checked with the plan. A policy search that finds no product-specific decision should likewise be reported as unresolved, rather than classified automatically as non-coverage.

Interpreting public financial information

The financial findings show that a displayed dollar amount is insufficient without an explanation of what it purchases and whether it remains valid. In this study, the Rejoyn promotional price had expired [25], the leva evaluation fee covered a clinical visit [26], and the larger leva amount came from an economic model [27]. These amounts cannot be compared as equivalent treatment prices. Rental terms [29], manufacturer-reported zero additional cost for eligible groups [31,32], and payment for professional management [20] also represent different financial arrangements.

For each quotation, clinicians, purchasers, and manufacturers should specify the product or service, treatment duration, included components, eligibility conditions, and expiration date. Professional fees and potential renewal charges should be identified separately. Manufacturer-reported patient costs should be checked against the patient’s actual benefit. Missing prices should remain unreported, rather than being treated as zero. The selected observations do not support an average market price, a representative price range, or an estimate of how often prices are publicly available.

Implications for payers and manufacturers

The observed documentation gaps extend these practical access and cost problems to the organizations publishing the information. They suggest specific improvements to public information. Payers should identify which plans and populations a policy applies to, link general criteria to product-specific decisions, and distinguish clinical eligibility from contractual benefit inclusion. They should make exception procedures and renewal requirements easy to locate and distinguish changes in clinical recommendations from administrative coding updates. Catalog administrators should identify whether a service requires a purchaser’s separate election. Manufacturers should distinguish payer-confirmed benefits from their own access statements and provide current product, fulfillment, and price information.

These are recommendations arising from the documents reviewed, not evidence that clearer communication alone would increase coverage or improve outcomes. The study did not determine whether any particular exclusion was clinically justified. That question requires assessment of the clinical evidence and the relevant benefit terms, rather than counting how often restrictive wording appears.

Priorities for future research

A national study should begin with a defined list of insurers, other organizations issuing coverage policies, and plans, then apply the same product and benefit searches to each. It should verify which plans use each policy, record product versions and policy dates, and use independent human extraction and adjudication. Enrollment should be used to estimate covered lives only when the applicable benefits and overlapping populations have been resolved. Price research needs a separate, consistently searched product list and comparable price definitions. Linking policy findings to authorization decisions, dispensing or activation, payment, and patient outcomes would help determine whether documented pathways translate into treatment access.

Limitations

The main limitation is source selection. Documents were chosen through purposive, adaptive searches rather than a national census or probability sample. Selection and coding were iterative, and policy positions were known during codebook development. The original query sequence and search-result screening counts were not retained, limiting exact reproduction of source discovery despite documentation of the included records. Restrictive policies may also be easier to discover through product searches. Although 21 of the 22 programs had at least one restrictive policy statement, that finding cannot be interpreted as the proportion of programs without coverage. The sensitivity analyses examine coding and product-definition choices but cannot correct potential selection bias. The remaining program had no extracted policy determination; absence of a determination does not establish non-coverage.

The study did not examine every policy or plan within the included organizations. Self-funded employer elections, member contracts, negotiated terms, and most exact formulary placements were unavailable. A document’s applicability to affiliated plans was not always established. Class-based criteria, changes in product labeling, and supervision requirements introduced additional uncertainty. Regulatory records did not establish that every marketed version was equivalent or that each product remained commercially available.

Finally, pricing and commercial availability were not checked uniformly across all programs. The financial findings therefore describe selected observations rather than the market as a whole. Because the financial records represent different economic quantities and eligibility conditions, they cannot support a pooled price estimate or a standardized comparison of treatment costs across products. No claims, authorization outcomes, dispensing, software activation, adherence, or patient outcomes were analyzed. The study cannot determine realized patient access, the number of covered patients, or the clinical and financial consequences of the policies.

## Conclusions

This cross-sectional health policy and market access study of 22 PDT programs found that regulatory authorization and available payment codes did not establish favorable medical-necessity determinations or routine benefits. Anthem’s product-specific restrictions were a prominent example, while conditional pathways in other policies, including its separate amblyopia guideline, showed why access must be assessed for the exact product and plan. The general-purpose-device-only analysis excluded two conditional-eligibility mappings and one class mapping with unresolved setting applicability, demonstrating that product definitions can materially change the findings. Public financial observations represented different economic quantities and could not support a meaningful pooled price estimate.

Before treatment is arranged, clinicians and access teams should verify the applicable product-specific policy, whether the patient’s plan adopts it, the benefit route, authorization requirements, and expected patient costs. Manufacturers and payers should distinguish regulatory status, clinical eligibility, benefit inclusion, and actual payment in their public information. These findings characterize selected documents and do not establish national coverage prevalence or realized patient access. Future research should combine systematic policy ascertainment and independent verification with plan-specific authorization, dispensing or activation, and payment data to determine whether documented pathways translate into treatment access.
